# Supplementary material for: Inhibitory Effects of Shikonin Dispersion, an Extract of Lithospermum erythrorhizon Encapsulated in β-1,3-1,6 Glucan, on Streptococcus mutans and Non-Mutans Streptococci
Source: Int J Mol Sci. 2024 Jan 16;25(2):1075. doi: 10.3390/ijms25021075 (PMC10816867; doi:10.3390/ijms25021075)
Supplement: Supplementary file 1 [file ijms-25-01075-s001.zip › ijms-2783762-supplementary.pdf]

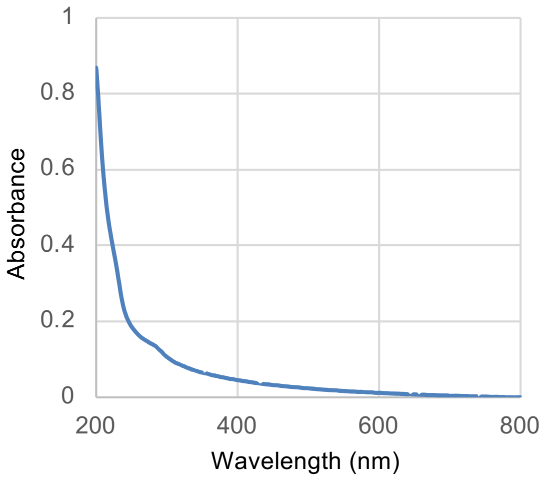

Figure S1. Absorption spectra of β-1,3-1,6 glucan solution.

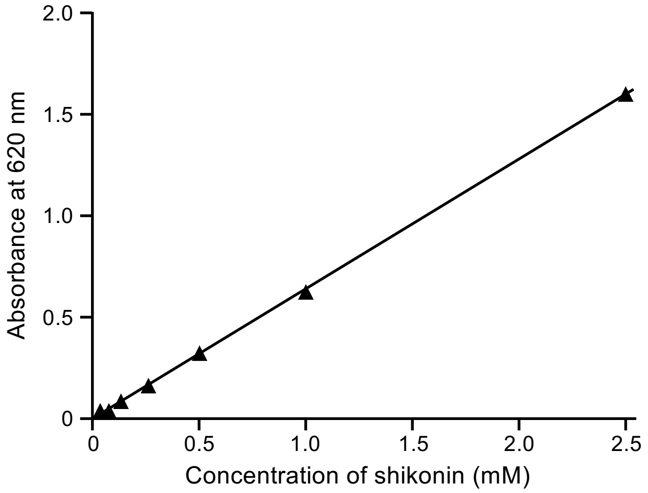

Figure S2. Calibration curve of shikonin dispersion in 1.0 M aqueous NaOH solution (*n* = 3, six technical replicates for each) .

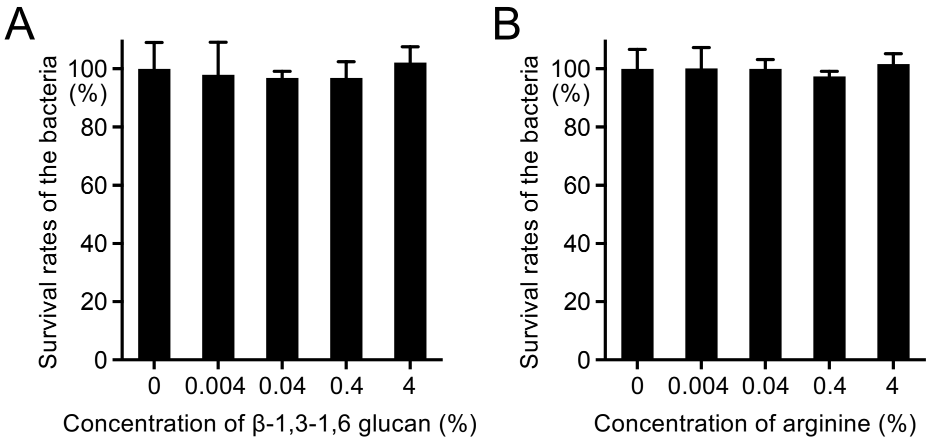

Figure S3. Effects of β-1,3-1,6 glucan and arginine solution on survival of *S. mutans* MT8148 suspended in phosphate-buffered saline (PBS). Inhibitory effect of β-1,3-1,6 glucan (A) and arginine (B) solution after 12 h on the survival of *S. mutans* MT8148. The concentration of β-1,3-1,6 glucan and arginine was calculated as 100% of the shikonin dispersion stock solution amount. Since 200 μM of shikonin corresponds to 4% of shikonin dispersion, the concentrations of shikonin (Figure 2) and β-1,3-1,6 glucan and arginine are equal in "w/v".

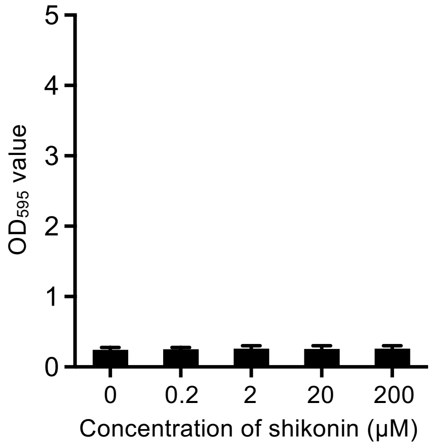

Figure S4. Effects of shikonin dispersion on biofilm formation by *S. mutans* MT8148 cultured in BHI broth without 1% sucrose.

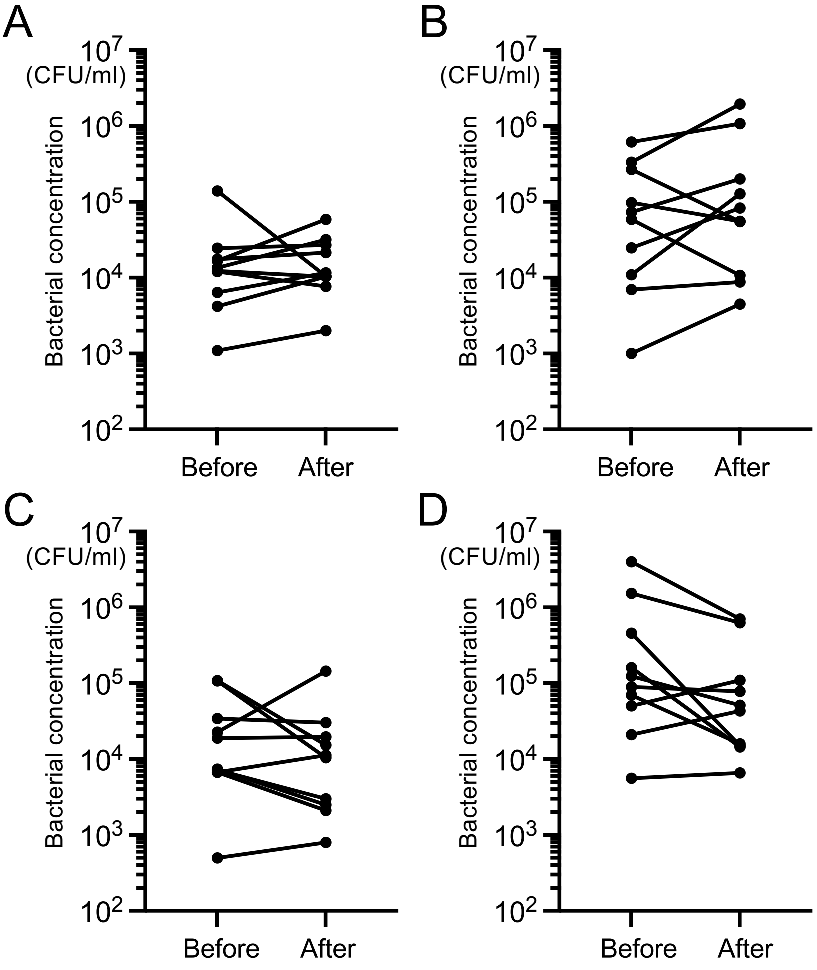

Figure S5. Changes in the number of *S. mutans* in saliva before and after using toothpaste with and without shikonin dispersion. The number of *S. mutans* (in CFU/ml) before and after brushing with toothpaste with no shikonin dispersion for 1 week in Group 1 (A) and Group 2 (B) and before and after brushing with toothpaste containing shikonin dispersion for 1 week in Group 1 (C) and Group 2 (D).
